# Intestinal Microbiota Modulation in Obesity-Related Non-alcoholic Fatty Liver Disease

**DOI:** 10.3389/fphys.2018.01813

**Published:** 2018-12-18

**Authors:** David Porras, Esther Nistal, Susana Martínez-Flórez, Javier González-Gallego, María Victoria García-Mediavilla, Sonia Sánchez-Campos

**Affiliations:** ^1^Institute of Biomedicine, University of León, León, Spain; ^2^Department of Gastroenterology, Complejo Asistencial Universitario de León, León, Spain; ^3^Centro de Investigación Biomédica en Red de Enfermedades Hepáticas y Digestivas, Madrid, Spain

**Keywords:** non-alcoholic fatty liver disease (NAFLD), obesity, intestinal microbiota, probiotics, prebiotics, polyphenols, fecal microbiota transplantation (FMT), physical exercise

## Abstract

Obesity and associated comorbidities, including non-alcoholic fatty liver disease (NAFLD), are a major concern to public well-being worldwide due to their high prevalence among the population, and its tendency on the rise point to as important threats in the future. Therapeutic approaches for obesity-associated disorders have been circumscribed to lifestyle modifications and pharmacological therapies have demonstrated limited efficacy. Over the last few years, different studies have shown a significant role of intestinal microbiota (IM) on obesity establishment and NAFLD development. Therefore, modulation of IM emerges as a promising therapeutic strategy for obesity-associated diseases. Administration of prebiotic and probiotic compounds, fecal microbiota transplantation (FMT) and exercise protocols have shown a modulatory action over the IM. In this review we provide an overview of current approaches targeting IM which have shown their capacity to counteract NAFLD and metabolic syndrome features in human patients and animal models.

## Non-Alcoholic Fatty Liver Disease (NAFLD)

NAFLD has emerged as the most common chronic liver disorder in Western countries, with an estimated prevalence of between 10 and 30% in USA and Europe and about 25% worldwide (Younossi et al., 2015). NAFLD development is strongly associated with obesity, type II diabetes and other features of the metabolic syndrome such as hypertension or dyslipidemia. In the case of obesity, the link with NAFLD is especially important since 75–92% of morbidity obese individuals present some degree of fatty liver (Fazel et al., 2016). This association implies that NAFLD will be more common in the near future due to the global epidemic of obesity, with more than 1,900 million people being overweight and more than 600 million obese in 2014 (World Health Organization, 2016).

Most NAFLD patients show a benign manifestation called steatosis defined as lipid accumulation in the liver. However, the disease can progress into more severe conditions such as non-alcoholic steatohepatitis, fibrosis, cirrhosis or even hepatocellular carcinoma (Tiniakos et al., 2010).

The pathophysiology of NAFLD is not yet fully understood with many factors involved in the progression of the disease. According to the multiple parallel hit hypothesis, all of them can act together simultaneously (Tilg and Moschen, 2010). Among these factors we found insulin resistance, oxidative stress, lipid metabolism alteration, inflammatory cytokines liberation, endoplasmic reticulum stress, intestinal dysbiosis or gut-liver axis activation (Buzzetti et al., 2016).

## Intestinal MICROBIOTA and NAFLD

The intestinal microbiota (IM) is a complex ecosystem where 10^14^ microorganisms coexists, mainly strict anaerobe bacteria though also facultative anaerobes, aerobes, small eukaryotes (fungi and protozoa) and viruses. The IM bacteria comprise two predominant bacterial phyla: *Firmicutes* and *Bacteroidetes* which reach as much as 90% of total bacterial population, although *Proteobacteria, Verrucomicrobia, Actinobacteria, Fusobacteria*, and *Cyanobacteria* are also represented (Sekirov et al., 2010). Colonization of gastrointestinal tract is believed to begin *in utero* and continues after birth until the early childhood (Francino, 2014) when the microbiome is relatively stable and specific of each individual (Zoetendal et al., 2008). Despite interindividual differences some authors have proposed the concept of enterotype as a core microbiome shared among the population, identifiable for a predominant bacterial genus: *Bacteroides* in the Enterotype 1, *Prevotella* in the Enterotype 2 and *Ruminococcus* in the Enterotype 3 (Arumugam et al., 2011). Later studies have questioned the existence of enterotypes or suggested a different classification but this attempt toward simplification could have an impact on clinical practice (Costea et al., 2018). IM acts as a live organ, carrying over protective, immune, metabolic and trophic functions (Miele et al., 2013), including non-digestible fiber fermentation and subsequent small chain fatty acids (SCFAs) production. The crosstalk between IM and the immune system, enforcing the symbiotic relationship between commensal bacteria and the host is noteworthy. This interplay modulates both adaptive and innate response to pathogens at the same time that prevents triggering immune activation in the absence of potentially harmful stimuli, thus maintaining a healthy state (Aron-Wisnewsky et al., 2013; Belkaid and Hand, 2014). In contrast, a disturbance of IM homeostasis with shifts in microbial composition, condition known as dysbiosis, may result in adverse effects for the host.

Intestinal microbiota imbalance has been documented in obese individuals (Ley et al., 2006) or with metabolic syndrome associated diseases (Qin et al., 2012; Mouzaki et al., 2013). A reduced proportion of *Bacteroidetes* and increased *Firmicutes* has been indicated as a main feature of obesity-related intestinal dysbiosis in humans (Ley et al., 2006), as well as a decrease of total bacterial diversity and richness (Turnbaugh et al., 2009; Le Chatelier et al., 2013). However, the statement of a higher *Firmicutes*/*Bacteroidetes* ratio as a hallmark of obesity is still controversial with most of the studies sustaining that, while others found no difference (Duncan et al., 2008) or even support the opposite (Schwiertz et al., 2010), although this can be attributed to methodological differences. Moreover, several interventions to reduce body weight, including dietary interventions as well as bariatric surgery consistently result in lower *Firmicutes* or increased *Bacteroidetes*, although there are still conflicting results (Seganfredo et al., 2017). Experience with animal models could bring some light into the association between high *Firmicutes*/*Bacteroidetes* ratio and obesity. First experiences with genetically obese (ob/ob) mice unveiled an overrepresentation of *Firmicutes* and reduction of *Bacteroidetes* (Ley et al., 2005). In nutritional models of obesity in mice using high fat diets (HFD), increased *Firmicutes*/*Bacteroidetes* ratio is a common outcome (Turnbaugh et al., 2008; Hildebrandt et al., 2009; Murphy et al., 2010) but it was suggested that this may be a feature of the diet rather than an obesity marker (Marchesi et al., 2016).

First studies linking NAFLD to dysbiosis reported an increased prevalence of small intestine bacterial overgrowth (SIBO) in NAFLD patients (Miele et al., 2013). Furthermore, it was suggested that dysbiosis in NAFLD may be related to different stages of the disease. In fact, dysbiosis in NASH patients differs from that with simple steatosis or with hepatocellular carcinoma development with a lower *Bacteroidetes* percentage associated to NASH (Mouzaki et al., 2013). This is in contrast with a later study where non-obese NAFLD patients exhibit lower *Firmicutes*/*Bacteroidetes* ratio than healthy controls, though this may be attributed to cofounding effect of body mass index (BMI). Moreover, this study reported a higher prevalence of Gram-negative bacteria and reduced diversity at phylum level in NAFLD patients (Wang et al., 2016).

Interestingly, ongoing down to the species level there is evidence of specific bacteria with potential implication in protection against obesity and NAFLD development. One of them is the mucin degrader, Gram-negative bacteria *Akkermansia muciniphila*. It was shown that *A. muciniphila* positively correlates with healthier metabolic status in mice and human studies with overweight and diabetic subjects (Dao et al., 2016; Plovier et al., 2016). On the other hand, some bacteria could be associated with the pathological state. *Helicobacter pylori* is frequently associated with diverse gastrointestinal disorders. Recent studies have found high prevalence of NAFLD in *H. pylori* infection positive individuals, suggesting the existence of a correlation between the presence of this bacteria and fatty liver development (Tang and Kumar, 2017).

## Mechanisms of DYSBIOSIS-Associated NAFLD

### Energy Harvesting From Diet

The role of dysbiosis in obesity is based on the increased capacity of gut microbiota from obese individuals to harvest energy from diet (Zhu et al., 2015). Germ-free mice (GFm)-based studies by Bäckhed et al. (2007) support this hypothesis, with reduced body weight gain in these animals vs. conventional raised mice following a high fat diet. Moreover, colonization of GFm with microbiota from conventional donors leads to a dramatic increase in body fat mass and development of insulin resistance (Bäckhed et al., 2004). Consistently, an interesting experiment with twins discordant for obesity resulted in greater body and fat mass in GFm receiving microbiota of obese twins (Ridaura et al., 2013). The greater efficacy on energy extraction from food has been related to metagenome of the intestine. Thus, microbiota of obese individuals may be enriched in bacterial genes implicated in carbohydrate, lipid and amino-acid metabolism (Turnbaugh et al., 2009). Genes related to cell membrane transport, mucus degradation, and production of toxins also seems to be overrepresented in the obese gut microbiome (Brahe et al., 2016). This leads us to think that not only the microbiome composition but also the microbial genome and its function determine the obesity prone state.

### The Gut-Liver Axis and the Immune System

**Figure 1:**
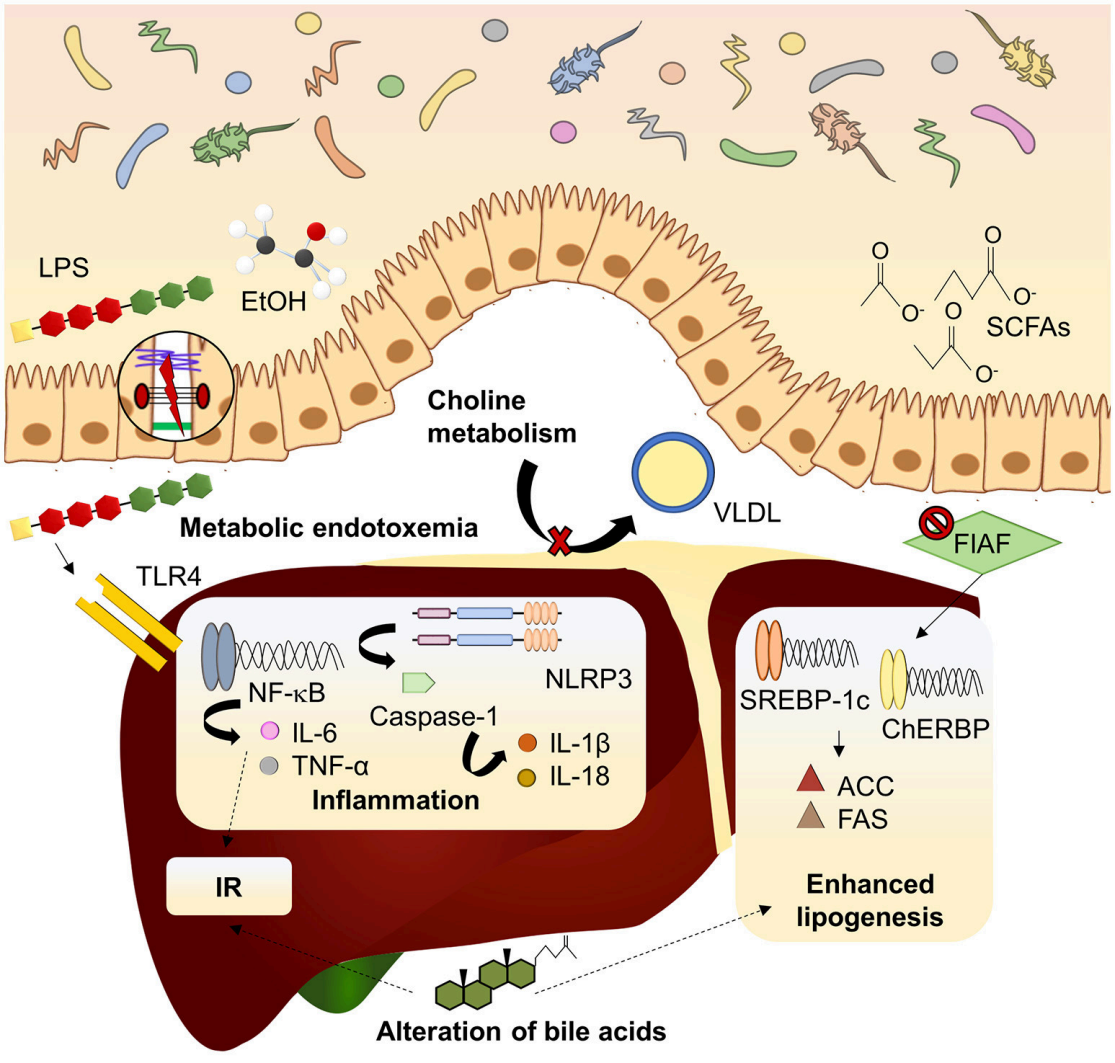
Mechanisms linking dysbiosis to NAFLD development. Dysbiotic gut microbiota is related to increased intestinal permeability and delivery of harmful substances (LPS, EtOH) to the liver, inducing inflammatory pathways mediated by PRRs. Inhibition of FIAF by IM promotes expression of lipogenic enzymes. Microbiota can modify bile acid pool in a mechanism associated to insulin resistance and lipogenesis enhancement. Choline metabolism is also affected by imbalanced microbiota, reducing lipid exportation through VLDL. ACC, acetyl-coA carboxylase; ChREBP, carbohydrate-responsive element-binding protein; EtOH, ethanol; FAS, fatty acid synthase; FIAF, fasting-induced adipocyte factor; IL, interleukin; IR, insulin resistance; LPS, lipopolysaccharide; NF-kB, nuclear factor kappa B; NLRP3, NOD-like receptor family, pyrin domain containing 3; SCFAs, short chain fatty acids; SREBP1-c, sterol regulatory element-binding protein 1c; TLR4, toll-like receptor 4; TNF-α, tumor necrosis factor α; VLDL, very low-density lipoprotein.

Beyond the role of the microbiome in the onset of obesity, mechanisms linking intestinal microbiota with NAFLD have been extensively studied in recent years (Figure 1). Major contribution of dysbiosis to NAFLD development takes place through the gut-liver axis which indicated the tight anatomical and functional relation between both organs (Vajro et al., 2013). Gut-liver axis involves components of the intestinal epithelium which constitutes the intestinal barrier, a tight monolayer of differentiated cells linked by the apical junctional complex (Catalioto et al., 2011). Compromised barrier function of the intestine, condition known as leaky gut, is another well documented manifestation linked to dysbiosis in NAFLD patients (Miele et al., 2009; Giorgio et al., 2014). Leaky gut allows translocation of potentially harmful bacterial products to the bloodstream, reaching the liver via the portal vein. Dysbiosis and subsequent impaired, barrier function of the intestine leads to the increase of gut derived toxins in systemic circulation constituting metabolic endotoxemia (Cani et al., 2007) which, in turn, contributes to the establishment of the chronic low grade inflammation state observed in obesity and NAFLD (Cani et al., 2012). This inflammatory state is strongly associated with the immune response mediated by the pattern recognition receptors (PRRs), mainly Toll-like receptors (TLRs), and nucleotide-binding and oligomerization domain (NOD)-like receptors (NLRs) in response to damage-associated molecular patterns (DAMPS) and pathogen-associated molecular patterns (PAMPs). TLR2, TLR9, and specially TLR4 have attracted the attention because of its well-known implication in NAFLD pathogenesis (Arrese et al., 2016). TLR4 is the principal sensor for lipopolysaccharide (LPS), the mayor component of the outer membrane of Gram-negative bacteria, and it is expressed in diverse liver cell lineages (hepatocytes and Kupffer and stellate cells). Recognition of LPS by TLR4 triggers of an inflammatory cascade which the last effector is the nuclear receptor kappa B (NF-κB), releasing a wide amount of proinflammatory cytokines. Circulating free fatty acids (FFAs) which are usually increased in NAFLD can also induce TLR4-dependent inflammatory pathway (Ferreira et al., 2015). Among the cytokines released, tumor necrosis factor (TNF)-α and interleukin (IL)-6 stands out for their contribution to insulin resistance development (Buzzetti et al., 2016). Otherwise, NLRs respond to its ligands forming the inflammasome complex. Most inflammasome studied in NAFLD is NLRP3 (NOD-like receptor family, pyrin domain containing 3), whose activation induces the recruitment of the adapter protein ASC and procaspase-1 (Szabo and Petrasek, 2015). Maturation of procaspase-1 by autocatalysis leads to the cleavage of proinflammatory cytokines such as pro-IL-1β or pro-IL-18. NLRP3 expression in the liver is increased in metabolic syndrome and NAFLD patients and its pharmacological blockade attenuates NASH development (Mridha et al., 2017). Intriguing, NLRP3-deficient mice present dysbiosis and this genotype is related to worse NAFLD progression (Henao-Mejia et al., 2012).

Gut-liver axis alteration is a good example of immune activation mediated by changes in gut microbiota composition, but crosstalk between microbiota, metabolic state and the immune system is rather more complex. For instance, a microbiota enriched in bacteria within the *Cytophaga*-*Flavobacter*-*Bacteroides* phylum has been related to the differentiation of IL-17-producing T-helper cells (Th17) in a TLR-independent signaling pathway (Ivanov et al., 2008). This mechanism could be relevant in fatty liver disease development as IL-17 is associated to inflammatory response and NAFLD progression (Tang et al., 2011). Moreover, dietary factors strongly determine this kind of response as some lipids in the diet (long chain fatty acids) induce the differentiation of Th17 while SCFAs determines an opposite action by promoting T regulatory cells proliferation (Honda and Littman, 2016; Ma et al., 2018). This points to a multidirectional interplay between microbiota, diet and immune system (Belkaid and Hand, 2014), thereby both atherogenic diet and HFD could induce NAFLD associated features in a mice model, but just the former trigger a systemic activation of the immune system in a mechanism related to different microbial composition between mice receiving each diet (Pindjakova et al., 2017).

### Impact on Lipid Metabolism

Lipid metabolism and transport are key processes in the establishing of NAFLD as steatosis arise from a deregulation of fatty acids input and uptake by the liver (Goedeke et al., 2018). Dysbiosis may enhance the steatotic process by the upregulation of enzymes implicated in *de novo* lipogenesis, mainly acetyl-coA carboxylase 1 (ACC1), and fatty acid synthase (FAS) (Bäckhed et al., 2004; Parnell et al., 2012). It is also remarkable the role of the fasting-induced adipocyte factor (FIAF). Synthesis of FIAF could be inhibited by enteric bacteria, increasing lipoprotein lipase activity and consequently triglyceride storage in the adipose tissue and the liver. Moreover, FIAF downregulation induces lipogenic enzymes expression through carbohydrate-responsive element-binding protein (ChREBP) and sterol regulatory element-binding protein 1c (SREBP-1c) (Leung et al., 2016).

### Metabolites Derived From Intestinal Microbiota

Some metabolites produced by gut microorganisms exhibit an antagonist role. On one hand, ethanol derived from alcohol producing bacteria like *Escherichia coli* may lead to hepatic injury in a similar way to what happens in alcoholic liver disease (Zhu et al., 2013). On the other hand, SCFAs are the main energy source for colonic epithelial cells, thus contributing to maintain intestinal integrity. Therefore, SCFAs supplementation had a positive effect in gastrointestinal diseases (Lu et al., 2016). However, some studies report elevated SCFAs in overweight patients (Schwiertz et al., 2010; Teixeira et al., 2013) pointing out that they are used as energy source increasing adipogenesis. This may be influenced by the specific SCFA; thus propionate can reduce *de novo* lipogenesis in the liver while acetate can be used as a lipogenic substrate (Tilg et al., 2016). In addition, lower butyrate and butyrate-producing bacteria are often related to metabolic disturbances (Brahe et al., 2013), suggesting that the specific pattern of SCFAs may be crucial for understanding their implication in obesity and NAFLD.

### Role of Bile Acids

A paradigmatic example of gut-liver communication is the enterohepatic circulation of bile acids. Apart from emulsification of lipids, bile acids act as signal molecules through the farnesoid X-receptor (FXR) (Wieland et al., 2015). Activation of FXR results in attenuation of lipogenesis and gluconeogenesis in the liver and improved insulin sensitivity. Given that gut microbiota can alter the bile acid pool and signaling properties, this is another interesting mechanism of dysbiosis-induced NAFLD development (Arab et al., 2017). Noteworthy, a gender-specific bile acids production pattern has been associated to FXR-dependent changes in intestinal microbiota in response to a Western diet, thus pointing to a potential causal link to the increased risk of NAFLD development in males (Jena et al., 2017; Sheng et al., 2017).

### Choline Metabolism

Finally, choline plays a crucial role in NAFLD. In fact, diets depleted in methionine and choline have been widely used in animal models to induced NASH (Almonacid-Urrego et al., 2012; Guzmán et al., 2013). Similarly, HFD-driven dysbiosis tends to reduce choline bioavailability promoting NASH development (Schnabl and Brenner, 2014). This is because choline is crucial for very low-density lipoprotein assembly and exportation of lipids from the liver. Moreover, microbiota may metabolize choline into toxic methylamines which enhance liver injury. Hence, dysbiosis promotes NAFLD through choline metabolism alteration in a dual way (Aron-Wisnewsky et al., 2013). Again, the relevance of this mechanism could be a gender issue as requirements for choline are lower in premenopausal women, who exhibit reduced risk of fatty liver development when they are subjected to choline deprivation (Fischer et al., 2007).

Taken all together, this evidence demonstrates that the microbiome defines metabolic state in humans, and makes a significant contribution to the establishing of obesity and NAFLD.

## Modulation of Intestinal MICROBIOTA

Although gut microbiota is relatively stable in adults, it is still susceptible to change. Environmental factors such as diet, exposure to toxic compounds or antibiotic consumption alter microbiome composition either in a positive or negative way. Therefore, it is possible to promote changes in microbiota composition to resemble a healthier profile.

**Figure 2:**
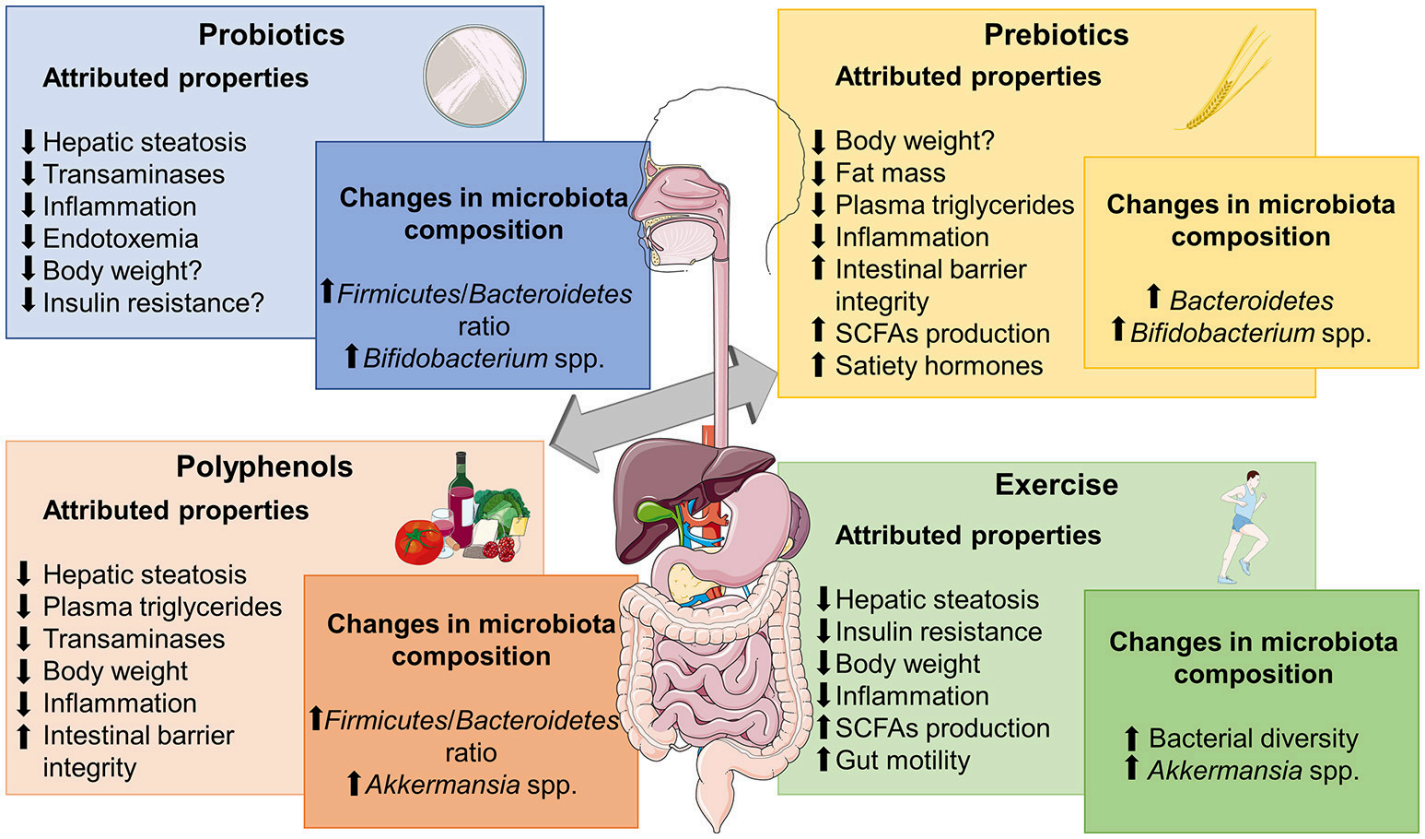
Metabolic effects frequently associated to different microbiome-based therapies for obesity-associated NALFD and relevant changes reported in microbiota composition. SCFAs, short chain fatty acids. The figure was made with use of Smart Servier Medical Art, licensed under a Creative Common Attribution 3.0 Unported License.

Due to the critical role of intestinal microbiota in NAFLD development, this approach may be suitable in clinical practice, so we should focus on current strategies that have shown modulatory capacity over dysbiosis and gut-liver axis activation, including substances with probiotic/prebiotic actions, Fecal Microbiota Transplantation (FMT) or physical exercise protocols (Figure 2).

### Probiotics

The use of live bacteria with potential health benefits for the host when provided in adequate amounts, namely probiotics, is a promising strategy to manage obesity-associated disorders. Bacteria belonging to *Lactobacillus, Streptococcus*, and *Bifidobacterium* genera are the most frequently used probiotics due to the growing body of literature supporting their health-promoting effects and because all of these integrate the normal human gut microenvironment (Iacono et al., 2011).

One of the first evidence of probiotics efficacy in the treatment of fatty liver was shown in a study conducted by Li et al. (2003) in which the administration of VSL#3 (a mixture of 8 lactic acid producer strains) to genetically obese mice improved liver histology, counteracted inflammation and showed a reduction of alanine aminotransferase (ALT) plasma levels. Following this experiment, many others have found effectiveness of probiotic treatment with different bacterial strains in animal models. This protective effect of probiotics in NAFLD is not yet fully understood but it has been proposed that antibacterial substance production, epithelial barrier function enhancement, and regulation of the immune system and subsequent intestinal inflammation may be implicated (Iacono et al., 2011).

Due to the nature of probiotics it is assumed that their beneficial effects are partially linked to IM modulation. Recent studies have assessed the modulation of microbiome in response to probiotics administration (Table 1). As mentioned above *Lactobacillus* species have been widely used in different animal studies despite the fact that some researchers have found an increased relative abundance of *Lactobacillus* in obese patients (Armougom et al., 2009; Million et al., 2012). Using HFD-fed mice, Kim et al. (2016) reported that probiotic supplementation with *Lactobacillus rhamnosus* GG can increase *Bacteroidetes* number, reduces *Firmicutes/Bacteroidetes* ratio and modulates dysbiosis. These results correlated with hepatic steatosis and dyslipidemic state amelioration, supporting findings of a previous work where *Lactobacillus rhamnosus* GG improved liver histology, modulating lipid metabolism, enhanced intestinal barrier integrity, and increased small intestine total bacteria concentration in a fructose-based NAFLD model (Ritze et al., 2014). *Lactobacillus* administration also achieved counteracting effects of a more aggressive model of NAFLD. In this way, *Lactobacillus casei* strain Shirota reverted reduction of *Lactobacillus* and *Bifidobacterium* genera abundance induced in response to a MCD diet while these contributed to diminish endotoxemia, inflammation and NASH development (Okubo et al., 2013). Distinct species may alter microbial composition in a particular way but with concordant results. Thus, *L. paracasei* CNCMI-4270 and *L. rhamnosus* CNCM I-3690 were equally capable of attenuating body weight gain, steatosis and insulin resistance but induced strain-specific changes in microbial structure in a HFD model (Wang et al., 2015). Authors also found metabolic syndrome amelioration due to *Bifidobacterium animalis* subsp. lactis I-2494 administration, concluding that each probiotic bacterial specie influence a particular set of functionally relevant microbial taxa.

**Table 1:** Modulation of microbiota by probiotics on *in vivo* models of NAFLD.

**References**	**Animal model**	**Probiotic**	**Time**	**Microbiota analysis**	**Main outcomes**
Kim et al., 2016	HFD-fed C57BL/6J mice	*Lactobacillus rhamnosus* GG (1 × 10^8^ CFU per mouse)	13 weeks	↑*Bacteroidetes* ↓*Firmicutes/Bacteroidetes* ratio (no significant)	↓Hepatic fat accumulation ↓Serum triglycerides and cholesterol ↓PPARγ, SREBP1, CACC, FAS, GPAT, CD36 and ApoB100 ↓IL-6 and IL12
Xue et al., 2017	HFHS-fed SPF SD rats	Probiotic mixture containing 0.5 × 10^6^ colony-forming units (CFU) live *Bifidobacterium infantis* and *Lactobacillus acidophilus* and 0.5 × 10^5^ CFU live *Bacillus cereus*	12 weeks	↓*E.coli* ↓*Enterococcus* ↑*Bifidobacteria* ↑*Lactobacillus* ↑*Bacteroides*	↓Liver inflammatory scoring ↓BWG Maintenance of tight junctions integrity and histology of the jejunum. ↑Occludin ↓LPS and TLR4 ↓TNF-α, IL-18 ↓ALT, AST, γ-GT ALP ↓TC, TG, LDL and FFA ↓HOMA-IR
Okubo et al., 2013	MCD-fed C57BL/6 mice	*Lactobacillus casei* strain Shirota (10^9^ CFU/day)	6 weeks	↑*Bifidobacterium* ↑*Lactobacillus* (*L. casei* and *L. reuteri*)	↓Hepatic lipid content, inflammation, ballooning and fibrosis ↓LPS ↓α-SMA and TIMP-1 ↓SREBP-1c and FAS ↓TNF-α ↓Colon inflammation (nuclear NF-κB/p65)
Wang et al., 2015	HFD-fed SPF C57BL/6J mice	*Lactobacillus paracasei* CNCM I-4270, *L. rhamnosus* CNCM I-3690, and *Bifidobacterium animalis* subsp. *lactis* CNCM I-2494	6 weeks	Strain specific modulation of key phylotypes belonging to: *↑Bifidobacterium* *↑Olsenella* ↑*Barnesiella* ↑*Allobaculum* ↑*Butyrivibrio* ↓*Desulfovibrionaceae* ↓*Oscillibacter* ↓*Clostridium* XIVa	↓Steatosis ↓BWG ↓HOMA-IR ↓Adipocyte size ↓CLS, MMP-12-positive cells and CD11c ↓Liver TNF-α (*B. animalis*) ↓LBP ↑Adiponectin (*B. animalis*)
Moya-Pérez et al., 2015	HFD-fed C57BL/6 mice	*Bifidobacterium pseudocatenulatum* CECT 7765	14 weeks	↑*Allobaculum* ↓*Lactobacillus* ↑*Bifidobacterium* ↓*Alistipes* ↑*Lachnospiraceae* ↑*Dorea* ↓*Bacteroides*	↓Steatosis ↓BWG ↓Cholesterol ↓Triglycerides ↑Insulin sensitivity ↓Epididymal adipose tissue ↓Endotoxemia/TLR4 ↓Liver and systemic inflammation
Cano et al., 2013	HFD-fed C57BL/6 mice	*Bifidobacterium pseudocatenulatum* CECT 7765	7 weeks	↑*Bifidobacterium* spp. ↓*Enterobacteriaceae*	↓Steatosis ↓BWG ↓Leptin levels ↓IL6 MCP1 ↑Insulin sensitivity
Kim et al., 2017	HFD-fed C57BL/6 mice	Kefir milk contained 9.84 ± 0.36 log CFU/ml of lactic acid bacteria and 7.23 ± 0.41 log CFU/ml of yeast	12 weeks	↓*Firmicutes/Bacteroidetes* ↑*Lactobacillus* ↑*Lactococcus* ↑*Candida* ↓*Proteobacteria* ↓*Enterobacteriaceae* ↓*B. fragilis*	↓NAS ↓BWG ↑PPARα AOX ↓IL-6 ↓Total cholesterol and LDL
Everard et al., 2014b	db/db mice	*Saccharomyces boulardii*	4 weeks	↑*Saccharomyces* and total yeast ↑*Bacteroidetes* ↓*Firmicutes* ↓*Proteobacteria* ↓*Tenericutes* ↓*Prevotella* ↑*Bacteroides*	↓Hepatic lipid content ↓BWG ↓Fat mass ↓CD11c, F4/80 and MCP-1 ↓Liver IL-1β ↓Serum IL-4, IL-6, IL-1β and TNF-α

*α-SMA, α-smooth muscle actin; ACC, acetyl-CoA carboxylase; ALP, alkaline phosphatase; ALT, alanine aminotransferase; AST, aspartate aminotransferase; BWG, body weight gain; CLS, crown-like structures; FAS, fatty acid synthase; FFA, free fatty acids; γ-GT, gamma-glutamyltransferase; GPAT, glycerol phosphate acyltrasferase; HOMA-IR, homeostasis model assessment of insulin resistance; IL, interleukin; LBP, lipopolysaccharide-binding protein; LDL, low-density lipoprotein; LPS, lipopolysaccharide; MCP-1, monocyte chemoattractant protein 1; MMP-12, matrix metalloproteinase-12; NAS, NAFLD activity score; NF-κB, nuclear factor kappa B; PPARγ, peroxisome proliferator-activated receptor gamma; SREBP-1c, sterol regulatory element-binding protein 1c; TC, total cholesterol; TG, triglycerides; TIMP-1, metalloproteinase 1; TLR4, toll-like receptor 4; TNF-α, tumor necrosis factor*.

It is noteworthy that a low proportion of *Bifidobacterium* has been observed in human studies associated with obesity and insulin resistance development (Brahe et al., 2016). It has been described in a HFD-based mice model that supplementation with *B. pseudocatenulatum* CECT7765 reduced hepatic steatosis and counteracted liver and systemic inflammation associated with decreased translocation of LPS to the bloodstream (Moya-Pérez et al., 2015). This effect correlates with partial restoration of intestinal microbiota especially in genera affected by the HFD belonging to the *Firmicutes* phylum (unclassified *Lachnospiraceae, Allobaculum*, and unclassified *Erysipelotrichacea*). *Bifidobacterium* abundance was restored to control levels in the probiotic treated group while Gram-negative bacteria enhanced by HFD (*Escherichia/Shigella* and *Desulfovibrionaceae*) were reduced. In a previous similar study the same strain also induced an increase in *Bifidobacterium* spp. in HFD-fed animals, while the opposite was true for *Enterobacteriaceae* (Cano et al., 2013). This shift in microbiota composition was associated with metabolic changes, thereby reducing body weight, fat accumulation in the liver, serum cholesterol, insulin resistance, and inflammatory cytokines.

It is not clear whether combination of probiotics could result in a synergistic effect. Positive results with VSL#3 and other *Lactobacillus*/*Bifidobacterium* mixes sustain this hypothesis. For example, intervention with a probiotic mix containing *Bifidobacterium infantis, Lactobacillus acidophilus*, and *Bacillus cereus* was capable of counteracting HFD-induced dysbiosis and related gut-liver axis activation along with the improvement of liver histology and inflammation. *Bacteroides*, bifidobacteria and *Lactobacillus* increased in the probiotic treated group, while *E. coli* and *Enterococcus* showed an opposite trend (Xue et al., 2017). Furthermore, kefir, a beverage containing up to 50 species of lactic bacteria, acetic acid bacteria and yeast improved obesity and steatosis in HFD-fed mice (Kim et al., 2017) by changing the intestinal microbiota. Thus, *Firmicutes*/*Bacteroidetes* ratio reduced in mice receiving kefir. Moreover, kefir administration increased the relative abundance of *Lactobacillus/Lactoccocus* as well as total yeast population, mainly *Candida*. The latter suggest that not only bacteria but also some yeast species may have probiotic effects. In this way, *Saccharomyces boulardii* reduced body weight, fat mass, inflammation and steatosis in obese diabetic (db/db) mice (Everard et al., 2014b). Metagenomic analysis of the cecal content of db/db mice revealed a notable increase in *Bacteroidetes* and a reduced proportion of *Firmicutes, Proteobacteria* and *Tenericutes*. Some genera associated to obesity and diabetes such as *Odoribacter, Ruminococcus*, and *Prevotell*a were also negatively affected, while others identified as beneficial like *Bacteroides* were increased.

In conclusion, therapeutic potential of probiotics has been extensively proved in animal models. HFD-based nutritional murine models remain the standard to evaluate the effect of probiotic over metabolic disturbances and hepatic steatosis. At the microbiome level, probiotics tends to induce changes in microbial composition toward a profile that is considered as beneficial, i.e., reduced *Firmicutes* to *Bacteroidetes* ratio or increased abundance of *Bifidobacterium* genus.

Some human studies have been carried out using probiotics in the management of NAFLD (Table 2). Despite promising clinical results, lack of microbiome based studies is the major weakness of these studies. Common outcomes of probiotics interventions include serum aminotransferases levels decrease and serum or liver lipid content lowering effects (Aller et al., 2011; Wong et al., 2013; Nabavi et al., 2014; Famouri et al., 2016). Some studies also found improvements in inflammatory markers. Anti-obesogenic effect of probiotics in humans is not yet fully accepted. Several groups found lower BMI in patients treated with probiotics (Alisi et al., 2014; Nabavi et al., 2014), others did not find significant changes. In this sense, results from the meta-analysis by Loman et al. (2018) confirmed that probiotic administration effectively reduces BMI and hepatic enzymes (ALT, AST, γ-GT), but their effect on lipid profile improvement did not reach significance. In addition, Sepideh et al. (2016) reported lower insulin and reduced HOMA-IR in the intervention group, constituted by NAFLD patients consuming two capsules a day of a probiotic mixture, supporting evidences from studies with diabetic patients (Yao et al., 2017).

**Table 2:** Clinical trials using probiotics as therapeutic strategy for NAFLD.

**Reference**	**Design**	**Probiotic**	**Time**	**Main outcomes**
Wong et al., 2013	Randomized controlled trial with biopsy proven NASH patients Probiotics (*n* = 10) Usual care (*n* = 10)	Lepicol probiotic formula (*Lactobacillus rhamnosus, Bifidobacterium bifidum, Lactobacillus acidophilus, Lactobacillus plantarum, Lactobacillus bulgaricus*)	6 months	Reduction in intrahepatic triglyceride content and ALT levels
Famouri et al., 2016	Randomized triple-blind trial in children with sonographicproven NAFLD Probiotic (*n* = 32) Placebo (*n* = 32)	Probiotic capsule containing *Lactobacillus acidophilus* ATCC, *Bifidobacterium lactis, Bifidobacterium bifidum Lactobacillus rhamnosus*	12 weeks	Improved ultrasonographicNAFLD indicator, reduction of ALT/AST, total cholesterol, LDL and triglyceride levels.
Aller et al., 2011	Randomized double-blind clinical trial in biopsy proven NAFLD patients Probiotic (*n* = 15) Placebo (*n* = 15)	*Lactobacillus bulgaricus* and *Streptococcus thermophilus*	3 months	Reduced ALT, AST, and γ-GT levels
Nabavi et al., 2014	Double-blind randomized clinical trial in ultrasonography proven NAFLD Probiotic (*n* = 32) Placebo (*n* = 32)	Yogurt containing *Lactobacillus acidophilus* La5 and *Bifidobacteriumlactis* Bb12	8 weeks	Decreased body weight and BMI, reduced ALT/AST, total cholesterol, and LDL levels
Alisi et al., 2014	Double-blind randomized clinical trial in NAFLD diagnosed obese children Probiotic (*n* = 24) Placebo (*n* = 24)	VSL#3	4 months	Improved fatty liver evaluation by ultrasonography, significant decrease in BMI, increase in circulating levels of GLP-1
Sepideh et al., 2016	Double-blind randomized clinical trial Probiotic (*n* = 21) Placebo (*n* = 21)	*Lactocare (Lactobacilluscasei, Lactobacillus acidophilus, Lactobacillus rhamnosus, Lactobacillus bulgaricus, Bifidobacterium breve, Bifidobacterium longum*, and *Streptococcus thermophilus)1g/day*	2 months	Reduced insulin, HOMA-IR, TNF-α, and IL-6

*ALT, alanine aminotransferase; AST, aspartate aminotransferase; BMI, body mass index; γ-GT, gamma-glutamyltransferase; GLP-1, glucagon-like peptide-1; HOMA-IR, homeostasis model assessment of insulin resistance; IL6, interleukin-6; LDL, low-density lipoprotein; TC, total cholesterol; TG, triglycerides; TNF-α, tumor necrosis factor*.

### Prebiotics

Prebiotics are defined as compounds that beneficially affect the health of the host by selectively stimulating the growth and/or activity of a limited number of bacteria (Everard et al., 2011). Many of the substances that respond to the prebiotic concept are nondigestible carbohydrates which provide a source of energy for commensal bacteria and are resistant to digestive enzymes reaching the distal gastrointestinal tract (Ojo et al., 2016). Within them, the most recognized are inulin-type fructans (inulin, oligofructose and fructooligosaccharides) and galactans (galacto-oligosaccharides) (Wilson and Whelan, 2017), although many others are under investigation, including lactulose, cellulose, resistant starches, hemicelluloses, gums, and pectins.

Prebiotic administration has shown a variety of actions concerning metabolic function in different studies. Reduced weight gain was found in several studies, in diet induced (Bomhof et al., 2014; Kumar et al., 2016; Steensels et al., 2017), in genetically obese rodents (Daubioul et al., 2000) and in overweight or obese humans (Parnell and Reimer, 2009) supplemented with prebiotic fiber. Alteration to appetite hormones release may be responsible for this anti-obesogenic effect (Cani et al., 2004; Hira et al., 2018). However, a meta-analysis of 26 human studies did not find any significant association between prebiotic consumption and body weight reduction (Kellow et al., 2014). Nevertheless, in some animal studies reduction of fat mass was achieved without significant body weight changes (Roberfroid et al., 2010). Treatments with prebiotics are frequently associated to fasting glycemia, glucose tolerance and insulin sensitivity improvement (Everard et al., 2011; Pachikian et al., 2013; Chan et al., 2016). Focusing on liver functionality, prebiotics have shown its capacity to modulate lipid metabolism. In this way, prebiotics are able to reduce plasma triglyceride levels (Correia-Sá et al., 2013) or limit its accumulation in the liver, preventing steatosis and liver injury in different animal models (Daubioul et al., 2002; Sugatani et al., 2006, 2012). Furthermore, improvement of insulin resistance, reduction in blood and hepatic triglycerides and prevention of steatosis have also been described for plant extracts enriched in fructans like Jerusalem artichoke in rats under a high fructose diet (Chang et al., 2014).

Prebiotic treatment has shown the ability to counteract inflammation. This effect may be related to the attenuation of serum LPS increase that occurs following dysbiosis (Cani et al., 2009; Neyrinck et al., 2012). LPS concentration reduction may be reached by the improved expression and activity of tight junction proteins like zonula occluden-1 (ZO-1) and occludin, preventing endotoxins translocation to the bloodstream (Delzenne et al., 2011). Furthermore, enhanced gut hormones release and endocannabinoid system blockade (Cani et al., 2009; Muccioli et al., 2010) are described mechanism exerted by prebiotics to promote gut barrier functionality, contributing to ameliorate inflammation and insulin resistance.

In line with the significant contribution of SCFAs to maintain intestinal health, a SCFAs production enhancement effect was described for some prebiotics, with special attention to butyrate (Everard et al., 2014a; Tochio et al., 2016). A synergistic action between butyrogenic species and acetate producers including species of *Bifidobacterium* genus in presence of oligofructose has also been documented (Falony et al., 2006). This is in accordance with repeatedly reported increase in *Bifidobacterium* spp. during fructooligosaccharides (FOS) supplementation (Cani et al., 2009; Meyer and Stasse-Wolthuis, 2009; Mao et al., 2015; Wang et al., 2017a). Other changes in microbial composition at phylum and genus levels were found in animal models of obesity after prebiotic consumption. Hence, 10 weeks of inulin/oligofructose (1:1) supplementation reversed the increase of *Firmicutes* and the reduction of *Bacteroidetes* observed in genetically obese rats, while at genus level leads to a dose-dependent increase in *Lactobacillus* and *Bifidobacterium* (Parnell and Reimer, 2012).

With regard to NAFLD, only a few studies have analyzed the effect of treatment with prebiotics or prebiotic-like compounds on gut microbiome and its impact in liver disease (Table 3). Beyond hepatic lipid content reduction, heterogeneity of models and substances used does not allow general conclusions. High fiber diet (Saha and Reimer, 2014), enzyme treated wheat bran (Kieffer et al., 2016), pectin (Fåk et al., 2015), and guar gum (Fåk et al., 2015; Janssen et al., 2017) interventions achieved body weight reduction, but no effect on obesity was found in the other treatments. In the study by Janssen et al. (2017) guar gum supplementation of a high fat/high cholesterol/high fructose also improved glucose tolerance and increased SCFAs production after 18 weeks of treatment. However, a surprising pro-inflammatory and pro-fibrotic effect were found in treated mice. At the microbiome level, three studies reported an increase in *Bacteroidetes* in line with the tend to lower *Firmicutes*/*Bacteroidetes* ratio in healthy subjects. As mentioned before, a bloom in *Bifidobacterium* is frequently associated to prebiotic supplementation; indeed high prebiotic fiber diet, guar gum, and FOS achieved this bifidogenic effect (Neyrinck et al., 2012; Pachikian et al., 2013; Saha and Reimer, 2014; Janssen et al., 2017). Other changes in microbiome composition were described in single studies and further investigation is needed to validate and interpret these findings.

**Table 3:** Modulation of microbiota by prebiotics on *in vivo* models of NAFLD.

**References**	**Animal model**	**Prebiotic**	**Time**	**Microbiota analysis after prebiotic administration**	**Relevant outcomes**
Pachikian et al., 2013	C57BL/6J mice fed an n-3 PUFA-depleted diet	0.25 g of FOS/day	16 weeks n-3 PUFA depleted diet + 10 days FOS supplementation	↑*Bifidobacterium* spp. ↓*Roseburia* spp.	↓Steatosis ↑Cecal weight ↑Insulin sensitivity ↑PPAR-α ↑GLP-1 ↓SREBP-2 ↓miRNA-33
Neyrinck et al., 2012	C57BL/6J mice fed a HFD	Arabinoxylan (10% w/w)	4 weeks	*Bacteroides-Prevotella* spp.*RoseburiaBifidobacterium animalis* spp. *lactis*	BWG Fat mass ZO-1, occludin Insulin resistance
Saha and Reimer, 2014	HFHS-fed rats	High fiber diet containing Inulin/oligofructose 216 (g/kg)	12 weeks High fiber or Control diet + 6 weeks HFHS + 4 weeks high fiber or control diet	↑Total bacteria ↑*Bacteroidetes* *↓Firmicutes* *↓Bacteroidetes/Firmicutes* ratio ↑Bifidobacteria ↑*Bacteroides* ↓*Clostridium leptum* ↓*Clostridium coccoides,* ↓*Clostridium cluster* I ↓*Clostridium cluster* XI ↓*Roseburia*	↓Total hepatic cholesterol ↓BW ↓Body fat ↑GLP-1 and peptide-YY
Kieffer et al., 2016	HFD-fed mice	Enzyme-Treated Wheat Bran (ETWB)	10 weeks	↑*Bacteroidetes* ↓*Firmicutes* ↑*Tenericutes* ↓*Verrucomicrobia* ↓*Proteobacteria* ↑S24-7 ↑*Rikellenaceae* ↑RF39 ↑*Ruminococcaceae* ↑*Adlercreutzia*	↓BWG ↓Liver TGs ↓Glucose (liver)
Fåk et al., 2015	Wistar rats fed with a HFD	Low methoxylated (LM) and high methoxylated (HM) pectin	3 weeks		↓Liver fat ↓BWG ↓Epidydimal fat ↓Plasma triglycerides (LM) ↑Total cecum SCFAs (HM)
		Low (LV), medium (MV) and high viscosity (HV) guar gum		*Bifidobacterium*	↓Liver fat (MV and HV) ↓BWG ↓fat ↓Plasma Cholesterol ↓Plasma triglycerides (LV and MV) ↑Total cecum SCFAs ↑Butyric acid (LV and MV)
Janssen et al., 2017	C57BL/6 mice fed with a high fat/high cholesterol/high fructose (HFCFD)	10% (wt./wt) guar gum	18 weeks	↓*Deferribacteres* ↓*Firmicutes* ↑*Bacteroidetes* ↑*Actinobacteria* ↑*Verrucomicrobia* ↑*Bifidobacterium* ↑*Prevotella* ↓*Lactobacillus* ↓*Oscillospira*	↓Steatosis ↓BWG ↓Liver weight ↑Cecum weight ↑Glucose tolerance ↑SCFAs ↑Inflammation ↑Fibrosis
Matsumoto et al., 2017	C57BL/6J mice fed a MCD	5% FOS in drinking water	12 weeks	↓*Clostridium* cluster XI ↓*Clostridium* subcluster XIVa ↑*Lactobacillales* spp.	↓NAS ↓Inflammatory cells infiltration ↓ALT ↓TLR4 (Kupffer cells) ↑SCFAs

*ALT, alanine aminotransferase; BWG, body weight gain; GLP-1, glucagon-like peptide-1; NAS, NAFLD activity score; PPARα, peroxisome proliferator-activated receptor alpha; SCFAs, short-chain fatty acids; SREBP-2, sterol regulatory element-binding protein 2; TG, triglycerides; TLR4, toll-like receptor 4; ZO-1, zonula occludens 1*.

A study by Matsumoto et al. (2017) in MCD-fed mice included insights from gut-liver axis preservation by FOS. FOS supplementation to MCD increased acetate and propionate and restored butyrate to control levels. Moreover, FOS contributed to preserve ZO-1 location in tight junctions, increased IgA secretion, and downregulated TLR4 expression in the liver. In agreement, Fåk et al. (2015) reported increased SCFAs production in HFD-fed rats as a result of guar gum and pectin administration, and found a specific profile for each compound and physicochemical state of the preparations. Moreover, physicochemical state of the compounds also differently affected microbiota composition and fat metabolism, pointing medium viscosity guar gum as the most effective preparation to prevent fat deposition in the liver in a mechanism associated to its bifidogenic effect and butyrate production capacity.

There are several studies which have tested the effects of prebiotics in obese and metabolic syndrome patients and performed metagenomic analysis. In a recent research with overweight children, oligofructose-enriched inulin administration significantly increased *Bifidobacterium* spp. and decreased *Bacteroides vulgatus* (Nicolucci et al., 2017). Microbiota composition modification was accompanied by reduced body weight gain and body fat along with attenuated IL-6 and triglyceride serum levels. Two studies in obese women subjected to inulin-type fructans administration showed concordant results in microbiome analysis pointing to increased representation of *Bifidobacterium* after the treatment (Dewulf et al., 2013; Salazar et al., 2015). Interestingly, in the study by Dewulf et al. (2013) changes induced by inulin-type fructans in *Bifidobacterium* and in *Faecalibacterium prausnitzii* negatively correlated with levels of LPS. The study by Salazar et al. (2015) went down to the species level and found that *B. adolescentis, B. longum* and *B. pseudocatenulatum* are the most affected *Bifidobacterium* species after prebiotic administration. Finally, a clinical trial in obese prediabetic individuals with galacto-oligosaccharides (GOS) corroborated the bifidogenic effect of prebiotics in obese humans (Canfora et al., 2017). However, no metabolic effects were reported. This is in conflict with a previous similar work where authors described reduced inflammatory markers, insulin resistance, total cholesterol and triglycerides linked to *Bifidobacterium* increase (Vulevic et al., 2013). Different dosage (15 g/day vs. 5.5 g/day) and study population (obese prediabetic vs. overweight and metabolic syndrome susceptible individuals) could be responsible for these contrasting results.

Evidence from human studies with prebiotics involving NAFLD patients is scarce. In a pilot study with NASH patients oligofructose administration (16 g daily for 8 weeks) achieved a reduction of AST levels and showed a tendency to lower ALT and triglycerides (Daubioul et al., 2005). However, lack of histological evaluation and microbiome-based analysis limits the conclusions of this study. A recent clinical trial with the same dosage of oligofructose for 24 weeks provides the first reports of histological improvement and changes in microbial composition in response to a prebiotic compound in NASH patients (Bomhof et al., 2018). Despite the small sample size, a significant reduction of steatosis and NAFLD activity score (NAS) were found in the prebiotic-treated group vs. placebo. Moreover, prebiotic administration resulted in increased *Bifidobacterium* and reduced *Clostridium* cluster XI relative abundance.

In addition, several clinical trials with synbiotics corroborated results obtained with prebiotics and probiotics administration alone (Malaguarnera et al., 2012; Ferolla et al., 2016; Mofidi et al., 2017). In the research conducted by Malaguarnera et al. (2012) the combination of *Bifidobacterium longum* and FOS with lifestyle modification exceeded benefits of lifestyle modification alone. Both treatments reduced NASH index, HOMA-IR, total cholesterol, LDL cholesterol, triglycerides, TNF-α, AST, and ALT serum levels. Moreover, supplementation with the synbiotic gave better results for HOMA-IR, LDL cholesterol, CRP, TNF-α, and AST. This greater efficacy of the synbiotic was associated with diminished serum endotoxin levels in this group. Moreover, Ferolla et al. (2016) also reported reduction of BMI and steatosis grade after inulin and guar gum intervention, despite the fact that there were no effects on SIBO or intestinal permeability.

Among the strategies to modulate intestinal microbiota, the administration of probiotics and prebiotics is the most developed so far, however there is still a lack in studies into humans. Ongoing clinical trials involving probiotics, prebiotics and synbiotics in NAFLD (Cho et al., 2018) could bring more light into this spot to make the step from basic research to clinical practice.

### Polyphenols

Vegetables polyphenols are known for their well described antioxidant and anti-inflammatory properties (González-Gallego et al., 2010). Polyphenols are classified according to their chemical structure in non-flavonoids, represented by stilbenes and phenolic acids, and flavonoids which constitutes a heterogeneous subgroup of compounds with a common C skeleton. Flavonoids are subsequently classified in flavanones, flavones, dihydroflavonols, flavonols, flavan-3-ols or flavanols, anthocyanidins, isoflavones, and proanthocyanidins.

Polyphenols are widely investigated both in *in vivo* and *in vitro* assays against different pathologies. Many of them have shown their capacity to counteract different features of metabolic diseases, including lipid metabolism alteration, insulin resistance, inflammation, and oxidative stress development (Pisonero-Vaquero et al., 2015b; Van De Wier et al., 2017). Furthermore, they are not completely digested in the gastrointestinal tract and suffer transformations by colonic microbiota when they reach large intestine, thus being considered prebiotic molecules (Gil-Cardoso et al., 2016). Moreover, the toxicity of the phenolic group gives them antibacterial activity and many of them may inhibit biofilm formation, pointing to another mechanism to influence microbiota composition (Espín et al., 2017). In addition, phenolic compounds metabolism into a large amount of low molecular weight bioactive metabolites and their dissemination in the organism make them capable of exerting vast effects in different organs including the intestine and the liver (Cardona et al., 2013).

Due to the contribution of inflammation and oxidative stress on its pathogenesis, besides the starring role of IM, NAFLD becomes a perfect target for polyphenol-based therapies. A large number of polyphenols as well as plant and fruits whole extracts with high polyphenol content have been tested with promising results. Quercetin, flavonoids from green tea, soy isoflavones, and rutin are the most studied flavonoids in NAFLD, along with silymarin, a mixture of flavonoids from *Sylbum marianum* whose major component is the flavonoid silybin (Pisonero-Vaquero et al., 2015a). Resveratrol is the most investigated polyphenol within stilbenes (Aguirre et al., 2014). Many of these studies were carried out in animal models but just the most recent include microbiome analysis to prove the prebiotic capacity of these compounds (Table 4). In addition, whole extract of grapes and different berries rich in polyphenols are effective in reshaping the microbiota of HFD-fed mice (Anhê et al., 2015; Baldwin et al., 2016; Heyman-Lindén et al., 2016).

**Table 4:** Modulation of microbiota by polyphenols on *in vivo* models of NAFLD.

**References**	**Model**	**Polyphenol treatment**	**Time**	**Microbiota analysis**	**Main outcomes**
Anhê et al., 2015	C57BL/6J mice fed a HFHS diet	Cranberry extract (200 mg/kg/day)	8 weeks	↑*Verrucomicrobia* ↑*Akkermansia*	↓BWG ↓Hepatic TG accumulation ↓Triglycerides and cholesterol (plasma) ↑Insulin sensitivity ↓HOMA-IR ↓LPS ↓Intestinal inflammation (COX2, TNF-α, NFκ/IκB ratio)
Heyman-Lindén et al., 2016	HFD-fed mice	20% (w/w) freeze-dried lingonberries	11 weeks	↓*Firmicutes/Bacteroidetes* ↑*Verrucomicrobia*↓*Proteobacteria* ↑*Parabacteroides* ↑*Odoribacter* ↑*Akkermansia*	↓Steatosis ↓Macrophage infiltration ↓BW ↑Cecum weight ↓Cholesterol ↓Glucose ↓SAA and LBP ↓TLR4 and EMR 1 ↑Occludin
Baldwin et al., 2016	HFD-fed mice	3% powdered grapes (w/w)5% powdered grapes (w/w)	10 weeks	↓*Desulfobacter* spp. ↑*Akkermansia muciniphilla* (not significant)	↓Hepatic TG content ↓Body fat (%) Improve ZO-1 localization
Porras et al., 2017	C57BL/6J mice fed with HFD	Quercetin	16 weeks	↓*Firmicutes/Bacteroidetes* ↑*Bacteroidetes*↓*Proteobacteria*↓*Desulfovibrio*↓*Helicobacter* ↑*Parabacteroides* ↑*Alkaliphilus* ↑*Akkermansia* ↑Total bacteria concentration	↓NAS ↓BWG ↓HOMA-IR ↓Triglycerides and FFAs ↓ALT ↓LPS etanol ↑SCFAs production ↑Claudin 1, Occludin and IAP ↓TLR4 ↓NLRP3 Caspase 1 ↓IL-6 ↓TNF-α and IL-6 ↓NF-κB/p65 ↓CYP2E1 ↓GRP78 CHOP
Masumoto et al., 2016	C57BL/6J mice fed with HFHS diet	Applepolymeric procyanidins (PPs)	20 weeks	↓*Firmicutes/Bacteroidetes* ↑*Akkermansia*	↓BWG ↓Liver weight ↓Visceral and subcutaneous fat ↓Glucose ↓Triglycerides and cholesterol ↓IL-6 TNF-α ↓LPS ↑ZO-1 and occludin ↓TLR4 and CD14
Roopchand et al., 2015	HFD-fed mice	1% Concord grape polyphenol extract absorbed to a soy protein isolate matrix	13 weeks	↓*Firmicutes/Bacteroidetes* ↑*Verrucomicrobia*↓*Proteobacteria*↓*Clostridia* ↑*Verrucomicrobiae* ↑*Verrucomicrobiaceae* ↑*Alistipes* ↑*Akkermansia* ↑*Akkermansia muciniphilla*	↓Hepatic lipid content ↓Liver weight ↑Cecum weight ↓Glucose tolerance ↓TNF-α and IL-6 (Blood and intestine) LPS ↑FIAF ↑Occludin ↑Proglucagon ↓Glut2
Feng et al., 2017	HFD-fed rats	Curcumin (200 mg/kg)	12 weeks of HFD feeding + 4 weeks daily doses of curcumin	↓*Tenericutes*↓*Ruminococcus*↓*Anaerotruncus* ↑*Lactobacillus*↓*Coprococcus*↓*Mucispirillum* ↑*Gordonibacter*↓*Helicobacter*	↓Hepatic lipid content ↓BWG ↓ALT/AST ↑ZO-1 and occludin ↓LPS and TLR4 ↓TNF-α and NF-κB
Van Hul et al., 2017	C57BL/6J mice fed a HFD	Grape Pomace extract	8 weeks	↑*Bacteroidetes*↓*Proteobacteria*↓*Desulfovibrio*↓*Lactococcus* ↑*Allobaculum* ↑*Roseburia*	↓Hepatic lipid content ↓Fat mass ↑Glucose tolerance ↓NEFAs
		Cinammon extract		↓*Peptococcus*

*ALT, alanine aminotransferase; AST, aspartate aminotransferase; BWG, body weight gain; CHOP, C/EBP homologous protein; COX-2, cyclooxygenase 2; CYP2E1, Cytochrome P450 2E1; EMR, EGF-like module-containing mucin-like hormone receptor-like; FFA, free fatty acids; FIAF, fasting-induced adipocyte factor; GLUT2, Glucose transporter 2; GRP78, glucose-regulated protein; HOMA-IR, homeostasis model assessment of insulin resistance; IκB, inhibitor of kappa B; IAP, intestinal alakaline phosphatase; IL, interleukin; LBP, lipopolysaccharide-binding protein; LPS, lipopolysaccharide; NAS, NAFLD activity score; NEFAs, non-esterified fatty acids; NF-κB, nuclear factor kappa B; NLRP3, NOD-like receptor family pyrin domain containing 3; SAA, Serum amyloid; SCFAs, short chain fatty acids; TG, triglycerides; TLR4, toll-like receptor 4; TNF-α, tumor necrosis factor; ZO-1, zonula occludens 1*.

In this way, supplementation of HFD or high fat/high sugar (HFHS) diet with polyphenols tends to lower the *Firmicutes/Bacteroidetes* ratio in rodent models, counteracting the effect of the diet (Etxeberria et al., 2015; Masumoto et al., 2016; Cheng et al., 2017; Guo et al., 2017; Porras et al., 2017). Furthermore, some bacterial genera and species seem to be more frequently influenced by these compounds. It is remarkable the frequent association of polyphenol administration with *Akkermansia* genus and *A. muciniphila* abundance (Anhê et al., 2015; Etxeberria et al., 2015; Roopchand et al., 2015; Baldwin et al., 2016; Heyman-Lindén et al., 2016; Masumoto et al., 2016; Porras et al., 2017). Considering that *Akkermansia* has a strong correlation with a healthy metabolic status, this observation enforces the protective role of polyphenols against NAFLD through gut microbiota modulation. The possible mechanism linking *Akkermansia* and metabolic effects of polyphenols is the maintenance of gut barrier integrity. *Akkermansia* is capable of preserving mucus layer preventing gut-liver axis disruption and endotoxemia (Gil-Cardoso et al., 2016). Thus, proteins involved in intestinal integrity maintenance are commonly modified by polyphenol treatments. Trans-resveratrol alone or in combination with quercetin significantly increased the expression of TJP (tight junction protein) 2 and occludin in HFD-fed mice (Etxeberria et al., 2015). This up-regulation effect was also found for TJP1 and occludin following the administration of procyanidins (Masumoto et al., 2016) or curcumin (Feng et al., 2017), and for claudin-1 and occludin after a quercetin treatment (Porras et al., 2017). Quercetin also tends to stimulate SCFAs production in *in vivo* models of metabolic syndrome and NAFLD (Etxeberria et al., 2015; Porras et al., 2017). SCFAs, especially butyrate, could be mediators of the polyphenol contribution to intestinal integrity. It is noteworthy that several butyrate-producing bacteria like *Faecalibacterium* and *Roseburia* are overrepresented in the microbiota of polyphenol-treated mice (Heyman-Lindén et al., 2016; Masumoto et al., 2016; Cheng et al., 2017; Guo et al., 2017; Liu et al., 2017; Van Hul et al., 2017).

In addition, beneficial effects of polyphenols can be attributed to inhibition of potentially harmful bacteria. As mentioned before, *Helicobacter pylori* has been related to NAFLD. There are studies reporting a decline in *Helicobacter* genus or *H. pylori* using flavonoids (Feng et al., 2017; Porras et al., 2017), flavonoid rich extracts (Chua et al., 2016; Asha et al., 2017), and another natural compounds (Zulueta et al., 2015).

Metabolic effects of polyphenols against NAFLD can be confirmed by human studies. Unfortunately, metagenomics analyses are lacking, except for a research that addressed the potential therapeutic use of red wine polyphenols in metabolic syndrome patients. In this clinical trial, 30 days of red wine or dealcoholized red wine were associated with increased detection of *Fusobacteria* and *Bacteroidetes* and reduced proportion of *Firmicutes* in fecal samples. Within *Firmicutes, Clostridium* was reduced while *Blautia coccoides, Eubacterium rectale, Faecalibacterium prausnitzii, Roseburia*, and *Lactobacillus* were increased after the treatment. With regard to *Bacteroidetes, Prevotella* detection was enhanced and *Bacteroides* diminished. Moreover, higher *Bifidobacterium* levels were found in the treated group along with a normalization of *Escherichia coli* representation (Moreno-Indias et al., 2016).

### Fecal Microbiota Transplantation

Fecal microbiota transplantation (FMT) was used as a therapy for the first time in present-day medicine in 1958 in order to treat pseudomembranous colitis due to *Clostridium difficile* infection (CDI) (Eiseman et al., 1958). Since then FMT has been widely used in CDI and it is now accepted as a successful treatment for recurrent CDI when antibiotic treatment fails. Moreover, after conflicting results (Moayyedi et al., 2015; Rossen et al., 2015), a recent study has shown clinical improvement following intensive-dosing multidonor FMT in patients with intestinal bowel disease (IBD) (Paramsothy et al., 2017) which, at least in part, involves dysbiosis as a pathogenic mechanism (Matsuoka and Kanai, 2015). Patients undergoing a FMT display a shift in microbiota composition characterized by an increase in microbial diversity due to the colonization of foreign bacteria, ideally with better metabolic functionality (Cohen and Maharshak, 2017).

Intestinal microbiota involvement in other pathologies points to FMT as an alternative treatment for a wide range of diseases, including extraintestinal pathologies. Metabolic disorders are possible future targets of FMT (de Groot et al., 2017). However, lack of clinical studies limits the evidence that support efficacy of this approach in obesity and associated metabolic syndrome. Just a short study in humans proved that transplantation of fecal microbiota from lean donors to metabolic syndrome patients resulted in the amelioration of insulin resistance which was related to increased butyrate-producing bacteria (Vrieze et al., 2012). However, a recent case report of a woman who quickly developed obesity after receiving FMT from an overweight donor (Alang and Kelly, 2015) suggests that the opposite is also possible.

Fortunately, studies with animal models provide useful information to determine FMT possibilities in metabolic diseases. For example, FMT from control diet-fed counterparts administered orally every third day for 8 weeks attenuates diet induced metabolic syndrome in Sprague-Dawley rats fed a high fructose diet (Di Luccia et al., 2015). Despite the fact that there is no effective measures against obesity, this treatment succeeded to ameliorate oxidative damage in the liver and skeletal muscle as well as systemic inflammation in a mechanism that involve reduction of plasma LPS. The improvement in metabolic condition was associated to lower representation of *Coprococcus* and *Ruminococcus* genera in the treatment group. Using a different approach, Rabot et al. (2016) did not find any effects in the onset of obesity in HFD-fed GFm after a single transplantation of feces from responder (R) and non-responder (NR) to the diet donors. They only reported a slightly improvement in glucose tolerance for NR receiver mice associated to a higher detection of *Bacteroidetes* in this group. Despite the fact that there is very different etiology, NAFLD shares some mechanism with alcohol liver disease (ALD), including the presence of dysbiosis. A recent study has shown that FMT prevents alcohol induced liver injury along with modulation of the IM (Ferrere et al., 2016). Feces from ethanol-resistant mice were administered to sensitive mice three times a week, reaching an amelioration of hepatic lesions. Microbiota from treated mice replicated that of resistant mice and both shared potentially beneficial genera of the *Bacteroidaceae* family: *Bacteroides, Parabacteroides, Prevotella*, and S24-7. Some studies have assessed the possibility to transfer metabolic improvement achieved after a prebiotic or polyphenol treatments trough FMT. Sung et al. (2017) found an improvement in glucose homeostasis and reduced fat mass in HFHS fed mice linked to changes in IM composition after 8 weeks of resveratrol supplementation. Feces collected from resveratrol treated mice were administered to another cohort of mice in three FMT experiments. Mice fed with the HFHS diet receiving FMT from resveratrol-treated mice showed improved glucose homeostasis in comparison with mice receiving feces from control mice and alterations of IM that resemble the profile observed in the donor group.

With regard to NAFLD, a study by Le Roy et al. (2013), in which microbiota from HFD-fed donors selected according to glycemia, inflammation and steatosis was transplanted to GFm, showed that the onset of obesity in response to a HFD in C57BL/6J mice is independent of the microbiota, in the same way as the experiment conducted by Rabot et al. (2016). However, they found lower fasting glycemia, HOMA index and NAFLD activity score (NAS) in the receiver of the NR microbiota. On the other hand, responder phenotype was associated with hepatic enzymes alteration and lipid metabolism disruption with increased *de novo* lipogenesis. This experiment demonstrated not only that intestinal microbiota is strongly associated with metabolic status and NAFLD, but also that NAFLD is a transmissible condition through intestinal microbiota transplantation. Recently, Zhou et al. (2017) carried out the first study of FMT in a diet-induced NASH model. Specific pathogen free (SPF) C57BL/6 mice were fed with a HFD for 16 weeks and given fresh feces from the control group daily for the latter 8 weeks. The transplantation resulted in reduced body weight, fat deposition, liver index and transaminase serum levels. FMT also restored intestinal integrity through recovering tight junction protein ZO-1 expression. This action over the intestine was related to increased butyrate production in the cecum and alleviation in endotoxemia. At hepatic level FMT clearly attenuated hepatic steatosis, lobular inflammation, and hepatocyte ballooning. Nevertheless, microbiota analysis showed opposite results to previous studies with HFD mice, reporting increased *Bacteroidetes* and reduced *Firmicutes* in the HFD group. Consistently, FMT treated mice display a shift toward the opposite, reducing *Bacteroidetes* to *Firmicutes* ratio. These authors also remarked the positive role of *Christensenellaceae* and *Lactobacillus* genera in metabolic disturbance prevention by FMT.

These results show the feasibility of FMT in the treatment of NAFLD. However, several issues on the procedure remain unanswered, such as parameters for donor selection, processing of feces, the use of single or multiple infusions or the usage of antibiotics previous to the transplant. Development of a non-invasive technique as the encapsulation of the donor microbiota to allow oral administration could spread this kind of treatment to a general audience (Delaune et al., 2018).

### Exercise

Lifestyle modifications are one of the traditional treatments in obesity related diseases. In NAFLD patients lifestyle change following dietary recommendations and more active behavior was associated with a moderate reduction of body weight, about 7–10%, which improved hepatic histology, decreasing NAS and its components (Vilar-Gomez et al., 2015). Apart from dietary interventions, physical exercise interventions are a valid way to reach weight loss. In human studies, physical exercise has demonstrated its ability to reduce cardiovascular events, improving metabolic state and counteracting many other features associated with obesity. With regard to liver disease, physical exercise is capable of modulating hepatic steatosis, improve insulin sensitivity or affect body composition independently of weight loss (Houghton et al., 2016). These changes are attributed to reduced expression of lipogenic enzymes, enhanced muscle fatty acids uptake, modulation of adipokines or attenuation of oxidative stress in the liver after exercise training (Ordonez et al., 2015).

Only few studies have considered that the effect of exercise on the metabolic state may be at least partially driven by intestinal microbiota modulation and no relation to NAFLD has been yet established. Modifications in intestinal microbiota have been found in rodent models under exercise protocols. Both voluntary wheel running (Matsumoto et al., 2008; Queipo-Ortuño et al., 2013) and forced running on a treadmill (Petriz et al., 2014) are effective in inducing changes in healthy animals. However these different approaches may differentially affect microbiome composition (Allen et al., 2015). In addition, development stage may influence plasticity of gut microbiota to changes induced by exercise. Thus, juvenile rats undergo larger modifications than adult individuals toward a leanness microbiota as evidenced by the increase in *Bacteroidetes* to *Firmicutes* ratio after 6 weeks of voluntary wheel running (Mika et al., 2015).

With regard to metabolic syndrome, Evans et al. (2014) provided evidences that 6 weeks of voluntary exercise may lead to a shift in microbiota composition along with weight gain prevention and improved insulin tolerance in mice fed with HFD. Supporting findings with probiotic/prebiotic administration, exercise tends to reduce *Firmicutes*/*Bacteroidetes* ratio as well as increase bacterial richness under HFD conditions. An analogous experiment by Campbell et al. (2016) confirms effects of voluntary exercise in HFD-fed mice on body weight gain along with increased insulin sensitivity, modulation of inflammatory state and enhanced secretion of satiety hormones. At the microbiome level, greater detection of *F. prausnitzii* in both lean and obese exercised groups as well as enrichment in *Allobaculum* and *Clostridium* genera are the most remarkable outcomes in this study. Furthermore, a forced running training protocol in genetically obese and hypertensive rats resulted in profound microbiota modification (Petriz et al., 2014). In this study, analysis of fecal samples showed an enhancement of bacterial diversity after training protocol. In contrast, these authors found increased relative abundance of *Firmicutes* with exercise in the three experimental groups (obese, hypertensive, and lean rats). At genus and specie levels *Lactobacillus* was increased in obese rats after exercise and six species (*Streptococcus alactolyticus, Bifidobacterium animalis, Ruminococcus gnavus, Aggregatibacter pnemotropica*, and *Bifidobacterium pseudolongum*) were more abundant in this group more than the others. The study into hypertensive condition is also relevant being hypertension a manifestation associated with NAFLD. Hypertensive rats displayed a particular microbiota and it was also susceptible to be modified by exercise, exhibiting greater *Allobaculum* and less *Aggregatibacter* and *Sutterella* after training. Finally, in a study with genetically diabetic and non-diabetic mice, forced running 5 days a week for 6 weeks correlated with lower *Bacteroides/Prevotella* spp. and *Methanobrevibacter* spp. (Lambert et al., 2015). Interestingly, exercise increased *Bifidobacterium* spp. in control mice but an opposite trend was found in diabetics, suggesting that previous metabolic state of the host influence the modulatory effect of the exercise protocol.

Welly et al. (2016) compared effects of exercise vs. caloric restriction in obesity-prone HFD-fed rats. Briefly, one group performed voluntary running while other remains as a weight-matched sedentary control by limiting the availability of food to mimic the weight loss achieved by exercise. They found that exercise exceeded benefits of weight loss alone, confirming that exercise effects against metabolic disturbances are independent of weight loss. Both treatments reduced adiposity, ameliorated inflammation and improved lipid profile but only exercise increased insulin sensitivity and achieved greater LDL reduction. These changes were related to microbiome modifications by exercise manifested by reduced relative abundance of two undefined genera in the *S24-7* and *Rikenellaceae* families and increased that of *Streptococcaceae* family in the exercised rats. Interestingly, the undefined genera of the *Rikenellaceae* family were positively correlated with liver triglycerides.

High intensity interval training (HIIT) seems to have similar or even greater metabolic effects than continuous aerobic training in diet-induced obese mice (Wang et al., 2017b) and in obese subjects (Wewege et al., 2017). Likewise, these effects can be related to changes in microbiota composition. According to Denou et al. (2016) in HFD-fed mice 6 weeks of HIIT performed 3 days per week induced shifts in the microbiota opposite to that promoted by diet, increasing *Bacteroidetes* as well as diversity within this phylum including a positive effect on *Bacteroidales* order and lowering *Firmicutes* in the distal gut. These changes correlated with improved insulin tolerance; however, authors did not find reduced body or fat mass in trained HFD-fed mice.

The impact of exercise on gut microbiota has also been shown in several human studies which identify a particular microbiota associated to individuals with divergent degrees of physical activity. Different microbiota composition was found between professional athletes and healthy controls, showing higher bacterial richness in athletes vs. controls (Clarke et al., 2014; Barton et al., 2017). Surprisingly, athletes present lower *Bacteroidetes* and greater *Firmicutes*. *Akkermansia muciniphila* increased both in athletes and in low BMI controls. Moreover, microbiota of active lifestyle women differs from those with a more sedentary pattern, with enhanced detection in the former of potentially beneficial bacteria like *Bifidobacterium* spp.*, Roseburia hominis, A. muciniphila*, and *F. prausnitzii* (Bressa et al., 2017).

In order to address the relationship between exercise-induced changes in microbiota and metabolic effects in the host, exercise impact on gut barrier should be considered. Several studies include evaluation of intestinal tissue after exercise in animal models. Exercise exerts protective effect on intestine epithelium counteracting the morphological changes associated to HFD and reducing inflammation (Campbell et al., 2016). This effect is sometimes attributed to alterations in gut motility by exercise that reduces intestinal transit time, but evidences are inconsistent. SCFAs also contribute to maintain intestinal health and their production was found to be significantly higher in professional athletes (Barton et al., 2017). In the experiment by Matsumoto et al. (2008) who described an increase in cecal n-butyrate concentration associated to the presence of two butyrate-producing bacterial species in the exercised group. The latter was also reported for Evans et al. (2014) who found that exercise significantly increased *Bacteroidales S24-7*, as well as butyrate-producing *Firmicutes* families *Clostridiaceae, Lachnospiraceae*, and *Ruminococcaceae*. Finally, intestinal health promoting bacteria like *A. muciniphila* are associated to exercise and active lifestyle in humans (Clarke et al., 2014; Bressa et al., 2017).

## Conclusion

Intestinal microbiota dysbiosis and related gut-liver axis activation are now accepted as a critical process in NAFLD development. Due to the absence of a gold standard treatment for fatty liver disease, development of novel therapeutic strategies is a priority to manage NAFLD patients. Different approaches have been made to modulate intestinal microbiota: pre/probiotics administration, FMT, and lifestyle modification including physical exercise interventions. All of these strategies lead to NAFLD and associated metabolic disturbances remission in *in vivo* models, but evidence from clinical trials is scarce. Moreover, only the most recent animal studies have carried out microbiome analysis to show that the results are linked to IM modulation. Furthermore, the findings obtained from these experiments need to be taken skeptically, due to the conflicting results. For this reason, it is too early to ensure the effectiveness of any of these methods. It is important to remark that different strategies are not mutually exclusive and maybe a synergistic combination of them could make the difference. In conclusion, intestinal microbiota modulation emerges as a potential therapeutic option for obesity-associated NAFLD. However, its application in clinical practice requires further investigation due to the lack in studies into humans.

## Author Contributions

DP wrote the manuscript. DP, EN, SM-F, JG-G, MG-M, and SS-C discussed the literature and figures, contributed to the intellectual input, and edited the manuscript. All authors read and approved the final version.

### Conflict of Interest Statement

The authors declare that the research was conducted in the absence of any commercial or financial relationships that could be construed as a potential conflict of interest.
